# PP03 Investigating Technological Strategies In The Hospital Setting: Insights From The Dutch Context

**DOI:** 10.1017/S0266462324001776

**Published:** 2025-01-07

**Authors:** Maria Pinelli, Emanuele Lettieri, Marcia Tummers, Janneke Grutters

## Abstract

**Introduction:**

Rapid advancements in technology are significantly impacting the healthcare system, and decision-making regarding technology adoption occurs at multiple decentralized levels within hospitals. National bodies seek to standardize this process, yet differing visions and strategies hinder centralization. This study explores the relationship between technological innovation and hospital strategies, focusing on scanning and assessment, aiming to assess the feasibility of centralized decision-making.

**Methods:**

To do this, we performed a qualitative analysis through 23 semistructured interviews in seven hospitals in the Netherlands, a country characterized by strong healthcare innovation and decentralization. We interviewed different actors involved in technological innovation, on different levels in the organization: CEOs, medical doctors, medical physicists or similar roles, and innovation managers. Ethics approval was obtained, and interviews were conducted, recorded, transcribed, and shared with participants for accuracy confirmation. Thematic analysis via grounded theory methodology and ATLAS.ti software generated insights on technological innovation’s relationship to hospitals’ strategies. Initial codes were refined into themes relevant to the research question.

**Results:**

Hospitals primarily aim to provide optimal patient care, with academic hospitals emphasizing research and education. Some hospitals aspire to be pioneers in adopting new technologies, while patient-centric healthcare is a shared goal. Technological strategies are not precisely designed in hospitals, being shaped by factors like people, financial constraints, or external environments. Hospitals’ scanning of technologies lacks systematization, and evaluations before and after technology adoption are not univocally performed. The need for systematic scanning and assessment practices is recognized by some interviewees, while others emphasize the importance of experimenting without the constraint of evaluation, perceiving it as a hurdle delaying innovation.

**Conclusions:**

Centralization could represent a benefit for hospitals, allowing them more streamlined decision-making, but it could also be perceived as a barrier. Involving hospitals’ stakeholders in centralization would be crucial to achieve it through a joint effort. Suggestions for future research could include focusing on a specific hospital, involving more stakeholders, and exploring other decentralized healthcare systems.

